# The NETting of pituitary adenoma: a gland illusion

**DOI:** 10.1007/s11102-022-01235-x

**Published:** 2022-05-26

**Authors:** Ken K. Y. Ho, Mônica Gadelha, Ursula B. Kaiser, Martin Reincke, Shlomo Melmed

**Affiliations:** 1 Garvan Institute of Medical Research and St Vincents Hospital, Sydney, Australia; 2 Universidade Federal do Rio de Janeiro, Rio de Janeiro, Brazil; 3 Brigham and Women’s Hospital and Harvard Medical School, Boston, MA USA; 4 Klinikum der Universität, Ludwig-Maximilians-Universität, Munich, Germany; 5 Cedars-Sinai Medical Center, Los Angeles, CA USA

Disease classification, fundamental to accurately reflecting critical knowledge of disease causes and consequences, underpins clinical care, service planning, payments, safety, and quality. Classification is subject to changes engendered by advances in the understanding of disease in health disciplines that reshape therapies to improve and maintain health.

The 5th Edition of the WHO Classification of Endocrine and Neuroendocrine Tumors brings changes to the 4th 2017 Edition in terminology for classification of neoplasms arising from anterior pituitary endocrine cells. A revised subclassification of some tumor subtypes is based on applying our knowledge of cell lineage tracing, employing both specific anterior pituitary hormone and transcription factor expression. Most striking, however, is the use of dual nomenclature of “pituitary adenoma/pituitary neuroendocrine tumor (NET)” in classifying pituitary neoplasms. Some of the chapter co-authors proposed 5 years ago that the term “adenoma” be replaced by “NET” because pituitary cells express markers similar to “NE” cells and pituitary neoplasms “bear some similarity to NETs in manifesting invasive and malignant behaviour” [1].

Care providers have questioned whether this pathology-based proposed change in classification is in fact evidence-based, and whether it is good news for patients with pituitary neoplasms. The answer requires a critical look at biology, taxonomy, prognostication, and clinical consequences of such a proposal.

## Biology

The WHO case for terminology change in the 5th Edition is explained in a commentary in *Endocrine Pathology* [2], which asserts that NET-like invasive and malignant behaviour is *frequent* among pituitary neoplasms. This claim is inaccurate and cannot go unchallenged. A “NET” label is a misrepresentation of the overwhelmingly benign clinical biology of pituitary neoplasms [3, 4]. The overall population prevalence of pituitary neoplasms is 10% or more. Local invasiveness occurs only in 1 in 2000 and malignancy in 1 in 100,000 such neoplasms [4]. Thus, “NET-like” behaviour occurs in a miniscule fraction, grossly mislabelling the overwhelming majority of pituitary neoplasms.

## Taxonomy

The proponents of nomenclature change to NET contend that pituitary cells are NE cells because they express NE markers such as synaptophysin, neuron-specific enolase, and somatostatin receptors. However, these markers are not specific to NE cells; they are also expressed in thyroid and adrenal neoplasms, which can also be invasive and occasionally malignant [5, 6]. Following the same reasoning, should these other endocrine neoplasms, including adrenal and thyroid adenomas, therefore not be classified as NETs? On the basis of taxonomy, as the difference between an endocrine cell and a neuroendocrine cell has not been defined, a name change confined solely to the pituitary without a critical review of overall endocrine gland taxonomy confuses and obfuscates the classification of endocrine tumors.

## Prognostication

Simply redressing adenomas as NETs does not change histopathology nor the prognosis of pituitary neoplasms. Unlike for the pituitary, bronchial and pancreatic NETs are graded according to prognosis. As correctly stated in the 5th Edition, a correlation between histological diagnosis and clinical behaviour of pituitary neoplasms is not established, predictive markers of malignant progression are not available, and no single histopathological marker reliably predicts pituitary tumor behaviour. There is simply no clinically relevant grading system available for pituitary neoplasms that is based on histology and which determines clinical outcome. *The clinical reality is that pituitary neoplasms are overwhelmingly benign regardless of semantic terminology.*

## Clinical consequence

In sharp contrast to the benign natural history of pituitary neoplasms, NETs are characterized by significant prognostic uncertainty. Cancer societies raise public awareness of NETs, citing metastases occurring in 50% of these patients at late diagnosis (https://nanets.net/education/about-nets); the Mayo Clinic patient education website states that NETs are “cancers that begin in specialized cells called neuroendocrine cells” (https://www.mayoclinic.org/diseases-conditions/neuroendocrine-tumors/symptoms-causes/syc-20354132). Selecting the right words to describe pituitary lesions is particularly important for patients and caregivers. Accordingly, disease labels such as cancer, nodule, and tumor play a significant role in patient decision making [7], especially because patients associate the word tumor with a malignancy [8]. There are also adverse consequences of changing disease classifications, including distorting perceptions of risk and prognosis as well as influencing management decisions [9]. Patients with low-risk disorders are less likely to benefit from treatments and more likely to experience detrimental adverse effects of interventions, anxiety caused by the disease label, and financial harm arising from additional and often unnecessary tests and treatments [9]. It is surprising that the patient perspective in such a relevant nomenclature shift has not been considered, that patient advocacy groups have not been counseled, nor have patient and public involvement representation been sought. There are indeed examples for taking patient perspectives into consideration in planned nomenclature changes for metabolic and endocrine diseases [10, 11]. The impact of a nomenclature change should not be underestimated, as demonstrated in a recent randomized controlled trial, in which the replacement of “papillary thyroid carcinoma” by “papillary lesion” impacted treatment decisions and anxiety levels [12].

In light of these clinical concerns, changes to disease labels require comprehensive evidence-based evaluation and rigorous challenge, as for other health care interventions such as indications for biopsy, or for performing costly imaging procedures. As responsible caregivers, we need to ensure that classification labels are based on evidence rather than opinion, and changes should not be made without due consideration of the potential impact across interrelated health care disciplines.

As an inseparable component of the endocrine system, the pituitary sits at the crossroads of several clinical management disciplines. Concerned with a proposal in 2017 from the Pituitary Club to reclassify pituitary adenomas as NETs [1] and the subsequent premature seeding of the term PitNET in the endocrine literature, the Pituitary Society convened an international workshop in 2019 to address the merit of the proposal. Experts in pituitary developmental biology, pathology, neurosurgery, endocrinology, and oncology, representing respective stakeholder professional societies and organisations, were invited. The IARC/WHO was unable to attend. This first interdisciplinary international workshop recommended that the term adenoma be retained as appropriate terminology for pituitary neoplasms arising from anterior pituitary cell lineages [6]. The recommendation and supporting evidence were communicated to the IARC/WHO for consideration in their preparation of the 5th Edition.

Nevertheless, the 5th Edition of the WHO Classification of Endocrine and Neuroendocrine Tumors retains adenoma in duality as transition terminology, likely foreshadowing a move to NET terminology in a future edition. If so, this is a disturbing development, signalling a decision oblivious to the Workshop concerns reflecting evidence-based conclusions of multidisciplinary experts that a nomenclature change distorts pituitary adenoma biology, confuses the classification of endocrine tumours, does not change prognosis, and engenders unnecessary social and health care anxiety.

Based on biology, taxonomy, prognostication and clinical consequences, the case for terminology change to NET has not been made, nor have the broad clinical consequences of a change in disease classification been considered. NET is a tunnel-view pathology label, imposing an unnecessary patient burden and liability on patients harboring an overwhelmingly benign pituitary neoplasm. There is an urgent need for a clinical classification system for pituitary neoplasms that meaningfully guides management outcomes, independent of ambiguous pathology-based terminologies. The classification of pituitary adenomas as PitNETs fails to fulfill this requirement.
